# Meal Quality of Entrées That Can Be Sold as Competitive Foods in Schools and Potential Impact of the Proposed USDA Rollbacks

**DOI:** 10.3390/nu12103003

**Published:** 2020-09-30

**Authors:** Juliana F.W. Cohen, Marlene B. Schwartz, Julien Leider, Lindsey Turner, Jamie F. Chriqui

**Affiliations:** 1 Department of Public Health and Nutrition, Merrimack College, 315 Turnpike Street, North Andover, MA 01845, USA; 2 Department of Nutrition, Harvard T.H. Chan School of Public Health, 677 Huntington Ave, Boston, MA 02115, USA; 3 Rudd Center for Food Policy and Obesity, Department of Human Development and Family Sciences, University of Connecticut, 1 Constitution Plaza, Hartford, CT 06103, USA; marlene.schwartz@uconn.edu; 4 Institute for Health Research and Policy, University of Illinois Chicago, Chicago, IL 60608, USA; jleide2@uic.edu; 5 College of Education, Boise State University, 1910 University Drive, Boise, ID 83725, USA; lindseyturner1@boisestate.edu; 6 Institute for Health Research and Policy and Division of Health Policy and Administration, School of Public Health, University of Illinois Chicago, 1603 W. Taylor St, Chicago, IL 60612, USA; jchriqui@uic.edu

**Keywords:** school, nutrition, competitive foods, school meals, legal epidemiology

## Abstract

The Healthy, Hunger-Free Kids Act strengthened competitive food standards (i.e., Smart Snacks), but an exemption allows reimbursable meal entrées that do not meet Smart Snack standards to be sold as “competitive entrées” on the same day they are served in the reimbursable meal, and the following day. Proposed rollbacks would enable these competitive entrées to continue to be sold on a third day, increasing the availability of competitive foods exempt from Smart Snacks standards. This study compared the Healthy Eating Index (HEI) scores of potential competitive entrées alone versus full reimbursable school lunches, and examined the nutritional characteristics of potential competitive entrées. Data were from a national sample of 1108 schools from the School Nutrition and Meal Cost Study. Linear regression models, accounting for school-level and state and district policy characteristics, found that HEI scores of competitive entrées were an average of 30 points lower than HEI scores of reimbursable lunches, with greater differences in small and rural schools. Less than 1% of common potential competitive entrees met Smart Snack standards, primarily due to higher sodium and saturated fat levels. The proposed rollbacks are estimated to potentially add approximately 662 mg of sodium and 3 g of saturated fat over three days (1103 mg sodium and 5 g saturated fat over a week) on average relative to Smart Snacks limits. Instead of increasing opportunities to sell competitive entrées, their sales should be further limited.

## 1. Introduction

In the United States, over 95% of public schools participate in the National School Lunch Program (NSLP) and the majority of schools sell competitive foods (i.e., individual foods and beverages sold during school hours through à la carte, vending machines, snack bars, school stores, and fundraisers) [1,2,3,4,5,6,7,8]. In the absence of strong nutrition standards, competitive foods often “compete” with school meals, with students often purchasing these items instead of the healthier school lunches [9]. In 2010, Congress passed the Healthy, Hunger-Free Kids Act (HHFKA) which enabled the United States Department of Agriculture (USDA) to update the nutrition standards for all foods sold in schools and to articulate the expectation that school districts update their local wellness policies which must include goals for nutrition education and physical activity, an assurance that school meal standards are aligned with federal standards, and a requirement that other foods sold in school meet the USDA’s Smart Snacks in Schools Standards [10,11]. The HHFKA strengthened the nutrition standards for both school meals and competitive foods (aka ‘Smart Snacks in School’) by aligning them with the concurrent Dietary Guidelines for Americans (2010), which emphasized a diet with more whole grains, less sodium, and a greater variety of foods [2,12,13]. For reimbursable lunches (i.e., meals that are eligible for at least partial reimbursement by the federal government), schools were required to offer five components (meat/meat alternative, grain, fruit, vegetable, and milk) and students were required to select three, including a fruit or vegetable as one of the required components.

Additionally, the school meal standards and the Smart Snacks standards both had progressive targets, with full implementation by the 2022–2023 school year and 2016–2017 school year, respectively. The updated standards included: (1) an increase in the quantity of whole grains offered (beginning with ≥50% whole-grain rich in the 2012–2013 school year and increasing to 100% by the 2014–2015 school year); (2) increased portion sizes for fruits and vegetables and the requirement that at least one of these components be included with every reimbursable meal; (3) an increased variety of vegetables offered throughout the week; (4) grade-specific limits on total calories for the meal; and (5) a phased reduction in sodium levels (the initial Target 1 implemented during the 2014–2015 school year, Target 2 during the 2017–2018 school year, and the final Target 3 during the 2022–2023 school year).

With the Smart Snacks standards, the USDA differentiated between ‘entrées’—defined as the main course of a meal consisting of a meat/meat alternative alone (with specific exclusions) or with a whole-grain rich food, vegetable, and/or fruit—and ‘snacks’, which were all other competitive foods [14]. By the 2016–2017 school year, the Smart Snacks standards required both entrées and snacks to be whole grain-rich OR have as first ingredient a fruit, vegetable, dairy product, or protein OR be a combination food with at least ¼ cup fruit/vegetable AND meet nutrition standards for calories, sodium, sugar, and fats (with differing levels for entrées and snacks). An important exemption, however, was that entrées sold as a part of a reimbursable school meal could also be sold as competitive foods on (a) the day the item was on the menu and (b) the next day—even if they did not meet Smart Snacks standards. For example, if pizza was an entrée in the reimbursable meal on Monday, pizza could also be sold individually as a “competitive entrée” on Monday and Tuesday, even if the pizza did not meet the Smart Snacks standards. This misalignment of the school meal and competitive food standards occurs because the NSLP standards are based on average nutrients for overall meals over the course of the week and not for individual food items.

Several studies have documented that the healthier school meal and competitive food standards have been successful; research indicates that the majority of schools are able to comply with the standards, and both participation rates and consumption of school meals have remained high [15,16,17,18,19,20,21,22,23]. However, in 2018 the USDA rolled back several aspects of the school meal standards, including delaying the Target 2 sodium standards, eliminating the Target 3 sodium standards, and removing the transition to 100% whole grains [24]. In 2020, the USDA proposed additional rollbacks to the standards. This included weakening the requirements regarding the portion sizes and variety for the vegetable categories at lunch, as well as enabling schools to serve vegetables (including potato products) instead of fruits at breakfast and reducing the portion size of fruit for breakfasts served outside the cafeteria [25]. Relevant to the competitive entrée exemption described above, the USDA also proposed adding an additional day to the number of days that non-compliant competitive entrées could be sold to students [25]. Using the pizza example above, the pizza from the reimbursable meal on Monday could be sold as a competitive entrée on Monday, Tuesday *and* Wednesday. The USDA cited that these rollbacks for school meals and competitive foods were based on concerns raised by program operators, including that healthier meals would result in increased food waste in cafeterias and that flexibilities were necessary to provide students with “nutritious” meals [24,26].

Currently, the extent to which typical competitive entrées (e.g., pizza, hamburgers, cheeseburgers, hot dogs, tacos, and breaded chicken/turkey nuggets) fall short of meeting the Smart Snacks standards is unknown. If the majority of these entrées meet Smart Snacks (even though they are not required to), this change may not be cause for concern. However, if competitive entrees are substantially less nutritious than Smart Snacks—or have a substantially lower nutritional value compared with the full, reimbursable meal—this proposed modification may have important implications for children’s diets and overall health [0].

The primary objective of this study was to evaluate the potential impact of the USDA proposed rollbacks by assessing the nutritional quality of potential competitive entrées (i.e., entrées from the school lunch that could be sold as competitive entrées) compared with reimbursable school lunches. Specific questions were: (a) what are the nutritional characteristics of potential competitive entrées; (b) how do the HEI scores differ between potential competitive entrées and reimbursable school lunches; (c) what school and state/district-level policy characteristics are associated with differences in HEI scores between potential competitive entrées and reimbursable school lunches; and (d) what is the potential impact of the proposed USDA rollbacks to entrées sold as competitive foods. The second objective of this study was to examine the differences between the Smart Snacks standards and the average nutritional characteristics of common meal entrées that are likely to be sold as competitive entrées.

## 2. Materials and Methods

### 2.1. Data and Design

Data from the nationally representative School Nutrition and Meal Cost Study (SNMCS) were used to assess the nutritional quality of reimbursable lunches and potential competitive entrées that were part of those lunches. Using a two-stage sampling approach in which schools were sampled within school food authorities (i.e., the entities that are responsible for the operations and administration of the local school meal programs), public (non-charter) schools in the 48 contiguous states and the District of Columbia that participated in the NSLP were recruited and *n* = 1282 out of 1284 agreed to participate. Data collection occurred from January through June of 2015 (2014–2015 school year, post-implementation of the updated school meal and Smart Snack standards). Additional study details, including information on recruitment and sampling methodology, have been previously published [15,27,28]. These data were linked with state laws and district policies from the National Wellness Policy Study [29,30], with de-identified data provided by Mathematica Policy Research to the University of Illinois Chicago for analyses. This study was deemed to “not involve human subjects” by the University of Illinois Chicago Institutional Review Board (protocol #2020-0448).

### 2.2. School Food Environments

To evaluate school meals, one week of school breakfast and lunch menus was assessed using an online menu survey filled out by school nutrition managers. Detailed information on all foods and beverages offered through the reimbursable meals was provided by the participating schools, including recipes and portion sizes; a total of *n* = 1207 completed data for school lunches (weighted response rate of 96%, accounting for raw sampling weights) [28]. This information was entered into the USDA’s Survey Net system (version 4.2) linked to the USDA’s Food and Nutrient Database for Dietary Studies (version 2011–2012) to assess nutrient content and linked to the USDA’s Food Patterns Equivalents Database and Food Pattern Equivalents Ingredients Database (version 2011–2012) to assess quantities relative to food groups, incorporating data from USDA’s Agricultural Research Service on common commercially prepared foods formulated specifically for use in school foodservice.

The current analyses examined the nutritional characteristics of the school lunch and items from the school lunch that would qualify as competitive entrées. Potential competitive entrées were identified based primarily on the major and minor food groups used for analyses of the Menu Survey data in the SNMCS report, with reference to data on the meal pattern contributions of each item from the Menu Survey and fat content for cheeses served alone to resolve ambiguities and as a cross-check [15]. Items identified in the data as combinations (e.g., a salad with crackers) were aggregated and treated as single items.

### 2.3. Healthy Eating Index and Nutritional Measures Regulated by Smart Snacks

Healthy Eating Index (HEI) scores, which evaluate a meal’s alignment with the Dietary Guidelines for Americans (DGAs), were calculated to assess the nutritional quality of reimbursable lunches and potential competitive entrées. HEI-2010 scores, which correspond to the 2010 DGAs, were used for the SNMCS as these were the concurrent guidelines when the study data were collected [31]. HEI scores assess nine adequacy components (dietary components to increase [i.e., total fruit, whole fruit, total vegetables, greens/beans, whole grains, dairy, total protein foods, seafood/plant protein, and fatty acids]) and three moderation components (dietary components to decrease [i.e., refined grains, sodium, and empty calories]). Each component has specific definitions and weights for minimum and maximum scores. The component scores are summed to derive a total HEI score (range 0–100), with higher scores corresponding to higher nutritional quality. This analysis used HEI-2010 scores for the reimbursable lunch previously computed for the SNMCS report based on average weekly menus prepared, and HEI-2010 scores for potential competitive entrées that were newly computed for this analysis [15]. Lastly, nutritional values that are regulated by the Smart Snack standards were computed for the potential competitive entrées (and compared with the standards): calorie content (≤350 calories), sodium content (≤480 mg), percent of calories from total fat (≤35% of calories), percent of calories from saturated fat (<10% of calories), and percent of weight from sugar (≤35% by weight).

### 2.4. Control Measures

School-level characteristics from the SNMCS and the National Center for Education Statistics were included as control measures in analyses examining differences in HEI scores between potential competitive entrées and reimbursable lunches [32]. These covariates included grade level (elementary, middle, high school), student population race/ethnicity (≥50% non-Hispanic White, ≥50% non-Hispanic Black, ≥50% Hispanic, and mixed), size (<500, 500–999 and ≥1000 students), and location variables including urbanicity (urban, suburban and rural) and Census region (West, Midwest, South, and Northeast) [33]. Lastly, the percentage of students eligible for free/reduced-price lunch (categorized by tertiles: ≤37.42%, >37.42–63.37%, and >63.37%) and a dichotomous indicator for whether the school offered universal free lunch (Yes/No) were included.

### 2.5. Policy Measures

Additional details regarding the methodology for collecting and coding state law and district policy have been previously reported [29,30]. State laws were compiled for all 50 states and the District of Columbia, and district policies were compiled for the public school districts where the SNMCS schools were located. For this analysis, two policy variables were included for each state’s laws and district’s policies to control for additional regulations that schools must adhere to in addition to the USDA’s school meal and Smart Snacks standards: (1) SM1, which measured the extent to which state law and district policies, respectively, assured that their school meal guidelines were not less restrictive than the USDA school meal regulations; and (2) NS12alc, which measured the extent to which the state law and/or district policy met the federal Smarts Snacks requirements for entrées and snacks sold à la carte in school cafeterias. Although school districts and schools have to comply with federal laws as a condition of reimbursement under the federal Child Nutrition Programs, research has shown that school nutrition environments and school food practices are stronger in states with laws mandating or providing a statewide framework [34,35]. SM1 was originally coded on an ordinal scale: 0 (no law/policy), 1 (should meet USDA school meal standards), 2 (must meet USDA school meal standards without specific details), and 3 (meets USDA school meal standards and defines the standards). NS12alc was originally coded on an ordinal scale for à la carte settings: 0 (no law/policy), 1 (recommends that foods/beverages sold meet Smart Snacks), 2 (meets Smart Snacks without details), 3 (meets Smart Snacks with standards defined), 4 (competitive food/beverage ban). For this analysis, both the SM1 and NS12alc variables were dichotomized into 0/1 variables with 0 = no law/policy or only suggested and 1 = required to meet federal standards (with or without details) or for NS12alc a competitive food ban.

### 2.6. Study Sample

Among the 1207 schools with school lunch data, 7 schools did not have any potential competitive entrée items on their menus. Among the 1200 remaining schools, a total of 104,292 distinct items prepared for reimbursable lunches were observed (counting items observed at different schools or days of the week separately), of which 24,420 were identified as potential competitive entrées. HEI scores and Smart Snacks nutritional measures were computed for each potential competitive entrée and then averaged across potential competitive entrées at each school to construct school-level measures. Out of the 1200 schools that had potential competitive entrées, 92 were excluded due to missing information (i.e., on or regarding universal free lunch status, student population race/ethnicity, and/or district policy data). Therefore, a total of 1108 schools located in 45 states and the District of Columbia and 365 school food authorities were included in all the present analyses. The percent of items meeting the standards was computed in the sample of 1200 schools that had potential competitive entrées. Due to differences in the sample employed, statistics shown herein may differ from those in the SNMCS report [15].

### 2.7. Data Analyses

Mean HEI-2010 scores for reimbursable lunches and potential competitive entrées were computed overall and by component, and the statistical significance of mean differences in HEI-2010 scores between potential competitive entrées and reimbursable lunches was computed using Wald tests. Mean values and inter-quartile ranges of nutritional measures regulated by Smart Snacks were also computed for potential competitive entrées. The differences between these mean values and the Smart Snack standards were calculated and multiplied by the additional potential number of days a competitive entrée could be served to approximate the potential impact of the proposed roll-backs to the standards: 3 days was selected to directly account for the proposed roll-backs and 5 days was selected as the maximum number of days a potential competitive entrée could be served during a school week (e.g., if an entrée was sold as part of the reimbursable school meal on a Monday *and* Wednesday or Thursday, that item could be sold as a competitive entrée every day that week). The association between the differences in HEI-2010 scores between potential competitive entrées and reimbursable lunches, overall and for specific components, and school-level characteristics and state and district policy measures was computed using linear regression models. Adjusted mean differences were computed from these models. In secondary item-level analyses, which were unweighted and not survey-adjusted, the nutritional characteristics of commonly served entrées that were considered more likely to be sold as competitive entrées (e.g., pizza, hamburgers, etc.) were compared with the Smart Snacks standards, and the percent of items meeting the standards was computed in the sample of 1200 schools that had potential competitive entrées. Analyses were conducted in Stata/SE (version 15.1, StataCorp LP, College Station, TX, USA; 2016) and took account of the survey design and weights.

## 3. Results

The analytical sample of schools was socioeconomically, racially/ethnically, and geographically diverse, and represented multiple grade levels and school sizes (Table 1). When examining state policies, 43.7% of schools were in a state with laws reinforcing federal school meal guidelines and 19.7% of schools were in a state with laws reinforcing or exceeding the federal Smart Snacks standards for à la carte items. For district wellness policies, 90.0% of schools were in a district with policies that included language reinforcing the federal school meal guidelines and 43.7% were in a district with policies that included language reinforcing or exceeding the federal Smart Snacks standards for à la carte items.

**Figure 1:**
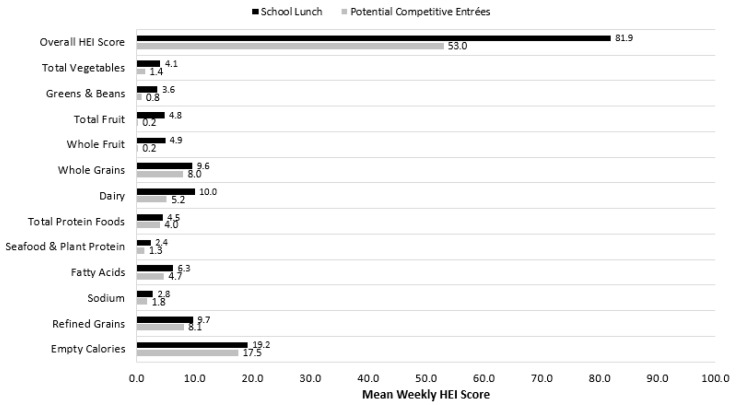
Survey-adjusted mean Healthy Eating Index (HEI)-2010 scores for school lunch vs. potential competitive entrée items alone. HEI-2010 scores for the school lunch were computed for each school based on the weekly average menu prepared. HEI-2010 scores for potential competitive entrée items were computed for each item and then averaged across all items prepared by the school in each school’s given target week. Mean HEI-2010 scores across analytical sample of 1108 schools from the 2014–2015 School Nutrition and Meal Cost Study (SNMCS), taking account of the survey design, are shown.

When examining the nutritional characteristics of the potential competitive entrées, these food items had on average 307.7 kcal and 700.5 mg of sodium (Table 2). Additionally, these foods had on average 37.4% of calories from total fat, 13.0% of calories from saturated fat, and 4.4% of weight from sugar. Average school-level HEI scores for both potential competitive entrées and school lunches were computed (Figure 1). On average, potential competitive entrées had an HEI score of 53.0; by comparison, school lunches had an average HEI score of 81.9. Similarly, HEI scores for potential competitive entrées were lower for all nine adequacy components and all three moderation components compared with school lunches. The statistical significance of average differences in school-level HEI scores for potential competitive entrées and school lunches was computed, and average differences overall and for each of the 12 components were significantly different from zero (*p* < 0.001).

Differences in the overall HEI scores between potential competitive entrées and school lunches were also examined by school- and policy-level characteristics (Table 3). Compared with schools in the lowest tertile for the percentage of students eligible for free/reduced-price lunches, schools in the medium tertile had significantly greater deficits in HEI scores (i.e., lower HEI scores for potential competitive entrées relative to school lunches; β = −1.57, 95% CI −3.08, −0.06). However, no significant differences were observed for schools in the highest tertile of students eligible for free/reduced-price meals relative to the lowest tertile. The deficit in HEI scores for competitive entrées was also larger for small schools compared with larger schools (β = −1.40, 95% CI −2.79, −0.01), although no significant differences were observed for medium size schools relative to larger schools. When examining location, schools in rural areas had significantly greater deficits in HEI scores compared with urban schools (β = −1.74, 95% CI −3.41, −0.08). Conversely, schools in the Northeast had significantly smaller deficits in HEI scores (i.e., higher HEI scores for potential competitive entrées relative to the scores of school lunches) compared with schools in the West (β = 3.14 [95% CI 1.08, 5.20]). No significant differences were observed for suburban relative to urban schools or schools in the South or Midwest relative to the West, nor were differences observed by school level, availability of universal free school lunches, or student population race/ethnicity. The presence of relevant state laws or district wellness policies was not associated with differences in overall HEI scores between potential competitive entrées and school lunches.

Differences in HEI scores for subcomponents between potential competitive entrées alone and the full school lunch were also examined by school- and policy-level characteristics. The subcomponents most relevant to the school meal standards (i.e., total vegetables, whole grains, protein foods, and sodium) are presented in Table 4. Total fruit was not included as the distribution was too skewed to be examined using linear regression. For the total vegetable subcomponent, there were significantly smaller deficits in HEI scores (i.e., higher HEI scores for potential competitive entrées relative to school lunches) for middle schools (β = 0.27; 95% CI 0.08, 0.47) and high schools (β = 0.23, 95% CI 0.03, 0.43) compared with elementary schools. The difference in total vegetable HEI scores also varied by the percentage of students eligible for free or reduced-price meals, with schools in the medium tertile for free/reduced-price lunch eligibility having greater deficits in HEI scores compared with schools in the lowest tertile (β = −0.30, 95% CI −0.53, −0.07). Additionally, the deficit in HEI scores for total vegetables was significantly greater in small schools (β = −0.42, 95% CI −0.72, −0.13) compared with large schools, as well as in rural schools (β = −0.46, 95% CI −0.80, −0.12) compared with urban schools. Rural schools also had significantly greater deficits in HEI scores for whole grains (β = −0.78, 95% CI −1.33, −0.23) although these schools had smaller deficits for sodium HEI scores (β = 0.63, 95% CI 0.04, 1.23) compared with urban schools. Lastly, differences in sodium HEI scores were observed by the race/ethnicity of the student population. Compared with schools with a majority non-Hispanic White population, schools with a majority non-Hispanic Black population and schools with a mixed population had significantly greater deficits in sodium HEI scores (β = −0.94, 95% CI −1.55, −0.33 and β = −0.57, 95% CI −1.07, −0.07, respectively). No differences by availability of universal free school lunches, Census region, or other school level-characteristics were observed for these subcomponents. When examining district wellness policies, the presence of policies that required meeting or exceeding the Smart Snack standards for à la carte items was associated with smaller deficits in HEI scores for sodium between potential competitive entrées and school lunches (β = 0.41, 95% CI 0.01, 0.81). When examining state laws, the presence of those that required meeting or exceeding the Smart Snacks standards for à la carte items was associated with smaller deficits in HEI scores for whole grains (β = 0.71, 95% CI 0.05, 1.37). No other differences in HEI scores were observed by state or district policies.

To compare the nutritional value of competitive entrées to Smart Snack standards, items most likely to be sold as competitive entrées were identified. These were: pizza, hamburgers, cheeseburgers, hot dogs, tacos, and breaded chicken/turkey nuggets. A total of 79.9% of these food items were within the calorie requirements (≤350 calories), but only 12.0% were in alignment with the sodium standards of ≤480 mg. Roughly a third (31.5%) met the total fat requirements (≤35% of calories) and only 19.8% met the standards for saturated fats (<10% of calories). All entrées met the sugar standards (≤35% by weight). Less than 1% were in alignment with all of these Smart Snack standards.

**Figure 2:**
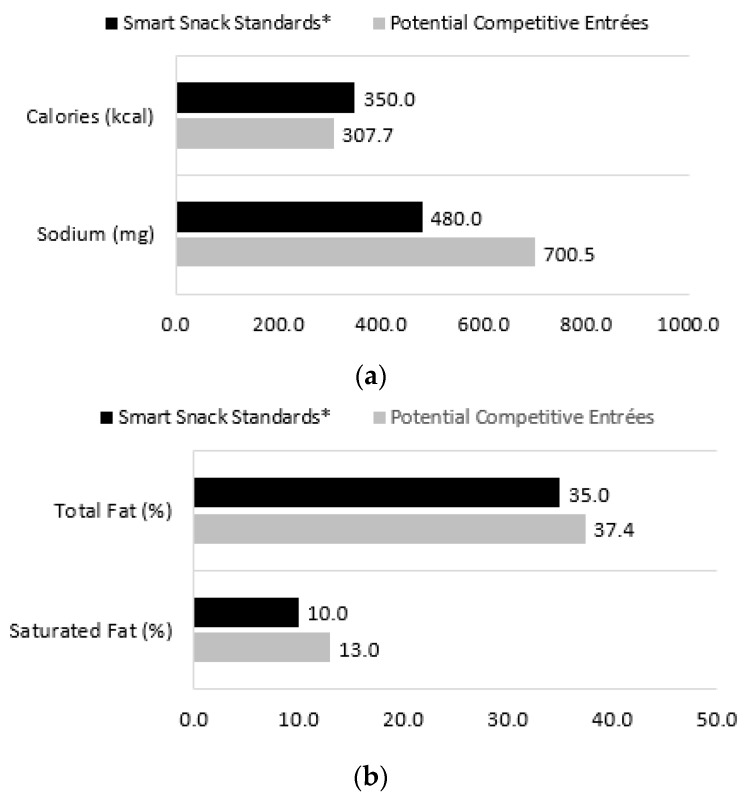
Average nutritional characteristics of potential competitive entrées compared with Smart Snacks limits. (**a**) Average nutritional characteristics of potential competitive entrées compared with Smart Snacks limits: Calories and Sodium; (**b**) Average nutritional characteristics of potential competitive entrées compared with Smart Snacks limits: Total Fat and Saturated Fat. Data on potential competitive entrées are school-level and take account of the survey design. School-level mean nutritional characteristics were computed across all potential competitive entrée items prepared in the given target week. Means were computed across schools. *n* = 1108 schools from the 2014–2015 School Nutrition and Meal Cost Study (SNMCS). * Values for Smart Snacks represent the maximum limit for nutrients.

The extent to which the average nutritional profile of potential competitive entrées (shown above in Table 2) exceeded Smart Snacks limits was used to approximate the potential impact of the proposed USDA rollbacks across a week. Mean sodium content (700.5 mg) was significantly higher than the Smart Snacks standard (480 mg; Figure 2a), and this would correspond to an excess of 662 mg of sodium over three days, or 1103 mg over five days (i.e., 700.5–480 mg × 3 days [or 5 days]). Mean percentage of calories from total fat (37.4%) exceeded the Smart Snacks limit (35%; Figure 2b), corresponding to an excess of about 2.5 g of fat over three days or 4 g over five days (based on the average calorie content of competitive entrées [307.7 kcal]). Mean percentage of calories from saturated fat (13.0%) also exceeded the Smart Snacks limit (10%), which would correspond to an excess of about 3 g of saturated fat over three days or 5 g over five days based on mean calorie content. The mean calorie content and percent of sugar by weight of potential competitive entrées fell below Smart Snacks limits.

## 4. Discussion

The proposed USDA rollbacks to the Smart Snack standards would allow more frequent sales of entrées as single items instead of as part of a balanced, reimbursable school lunch. The current findings suggest that this change would negatively impact the nutritional environment of school cafeterias, and potentially undermine the progress that has been made in the years following the updated standards from the Healthy, Hunger-Free Kids Act. The average HEI scores of the potential competitive entrées were nearly 30-points lower than reimbursable school lunches. This was due in part to the high sodium and saturated fat levels in the potential competitive entrées. Additionally, while the NSLP requires that milk, fruits, and/or vegetables are included as part of the reimbursable meal, these are not required components for competitive entrées, which also led to meaningful differences in the HEI scores.

Differences in the HEI scores between potential competitive entrées and school lunches were observed to vary by school characteristics, including the percent of the population eligible for free/reduced-priced meals, school size, and urbanicity. Larger deficits in the HEI scores for potential competitive entrées relative to school lunches were observed in both small and/or rural schools compared with large and urban schools, respectively. These findings suggest that these schools, which tend to have the fewest resources, may benefit by more support, such as greater access to healthier, low cost items in combination with stronger district wellness policies. Interestingly, schools in the middle tertile for eligibility for free/reduced price meals also had greater deficits in HEI scores compared with higher income schools, while no significant differences were observed when comparing the lowest and highest income schools. This may be in part due to resources available to school districts serving high proportions of students from economically-disadvantaged households, such as the ability to use the USDA’s Community Eligibility Provision to provide universal free meals, and the typically greater participation rates in school meals overall at these schools, which enhances the fiscal stability of district food service programs [36,37]. When examining district wellness policies, those that required meeting or exceeding the Smart Snack standards for à la carte items were associated with smaller differences between the HEI sodium scores of potential competitive entrées and reimbursable school lunches. For state laws, those that required meeting or exceeding the Smart Snack standards for à la carte items were associated with smaller differences between the HEI whole grains scores of potential competitive entrées and reimbursable school lunches.

The current findings also highlight the degree to which potential competitive entrées fail to meet Smart Snacks standards. Less than 1% of the commonly served, popular entrées (including pizza, burgers, tacos, hot dogs, and chicken nuggets) were fully compliant with all standards. The nutrients of greatest concern were the high levels of sodium and saturated fat, especially among the commonly served entrées where the majority exceeded both the sodium and saturated fat recommendations. The higher sodium levels were typically a greater issue in districts without strong wellness policies. While the proposed USDA rollbacks only add one additional day to the frequency of non-compliant foods being sold, serving one of these entrees twice a week in the school lunch would allow selling it as a competitive entree every day of the week. Therefore, the proposed rollbacks may undermine the original intent of the HHFKA as the foods available in schools will have diminished alignment with the Dietary Guidelines for Americans and will potentially reduce the variety of foods consumed by students. This may also lead to conflicting messages to students about what constitutes healthy, balanced meals, as well as have important dietary and health implications as many children consume up to half their daily energy intake at school [38]. When children consume one of these competitive entrees, they are receiving an estimated additional gram of saturated fat and 220 mg of sodium per day on average (when compared to a compliant Smart Snack), and are missing out on access to fruits or vegetables (when compared with a reimbursable school lunch). While the portion sizes of the individual potential competitive entrées are the same as those served as part of the reimbursable school meals, students are also able to purchase multiple competitive entrées at lunch (e.g., two or three pizza slices), which would lead to even greater saturated fat and sodium intakes. Increasing the frequency that children can consume competitive entrees risks increasing their risk of negative health outcomes over time. Previous research among children has found that a Dietary Approaches to Stop Hypertension (DASH)-style diet (which includes fruits and vegetables and lower quantities of sodium and saturated fats) is associated with lower systolic blood pressure and reduced risk of unhealthy weight gain [39,40]. Among children with diabetes and/or metabolic syndrome, these healthier dietary patterns have also been associated with lower blood pressure as well as a reduction in other cardiovascular disease risk factors including lower cholesterol and inflammatory marker levels [41,42,43,44]. Finally, recent research has also found an association between consumption of the healthier school meals and reduced consumption of solid fats and added sugar as well as a decrease in the prevalence of overweight and obesity [45,46].

This study has several limitations. First, because the SNMCS does not provide detailed information on the competitive entrées actually served, this analysis only examined potential competitive entrées based on the meals served in each school. However, secondary analyses examined some commonly served entrées, which are likely to be served more regularly as competitive entrées given the popularity of these dishes. Additionally, the analyses assumed only one competitive entrée was selected per child and therefore these are conservative estimates of the impact of the proposed rollbacks. Future studies should directly measure students’ selection and consumption of competitive entrées to determine if similar results are observed. Furthermore, the regression analyses were cross-sectional and cannot establish causality. Finally, as with all studies of this type, this study is subject to error in reporting by school nutrition managers and human error in data entry/nutritional coding; but the best available nationally representative data were used for this purpose. This study was strengthened by its large, nationally representative sample of schools and menus.

## 5. Conclusions

This study suggests that the proposed increase in the frequency that entrées from reimbursable school meals can be sold individually as non-compliant competitive entrées may lead to children having greater access to substantially less healthy lunch options at school that are in poorer alignment with the Dietary Guidelines for Americans. Further, the differences in the HEI scores between the potential competitive entrées and complete school meals were greatest in small and rural schools, suggesting that these changes could exacerbate existing disparities in the quality of food available to students [47,48]. Finally, the cumulative impact of providing these less healthy meal options more frequently may have important implications for children’s diets and overall health. Rather than weaken the competitive entrée standards, policy makers at the district, state, and federal levels should consider further strengthening the meal program standards by removing exemptions for non- compliant competitive entrées.

## Figures and Tables

**Table 1:** Analytic sample characteristics.

Variable	% (95% CI)
State Law Requirements	
Schools must follow federal school meal guidelines	43.7 (38.3–49.3)
Schools must meet or exceed federal Smart Snacks standards for a la carte items	19.7 (15.6–24.5)
District Wellness Policy Requirements	
Schools must follow federal school meal guidelines	90.0 (86.2–92.9)
Schools must meet or exceed federal Smart Snacks standards for a la carte items	43.7 (38.2–49.3)
School Level	
Elementary school	59.7 (57.3–62.0)
Middle school	18.2 (16.6–19.8)
High school	22.2 (20.4–24.1)
School Offers Universal Free Lunch	
Yes	19.2 (15.4–23.7)
No	80.8 (76.3–84.6)
Race/Ethnicity of Students in School	
≥50% Non-Hispanic White	63.2 (58.1–68.1)
≥50% Non-Hispanic Black	9.8 (6.9–13.8)
≥50% Hispanic	13.2 (10.2–16.9)
Mixed	13.7 (10.9–17.2)
Free/Reduced-Price Lunch Eligibility Rate Tertiles	
Low (0.00–37.42)	28.5 (24.3–33.1)
Medium (>37.42–63.37)	32.8 (28.7–37.3)
High (>63.37–100.00)	38.7 (34.0–43.6)
School Size	
Small (fewer than 500 students)	47.5 (43.0–52.0)
Medium (500 to 999 students)	40.1 (35.8–44.5)
Large (1000 or more students)	12.5 (10.6–14.5)
School Urbanicity	
Urban	22.6 (18.7–27.1)
Suburban	43.6 (38.7–48.6)
Rural	33.8 (29.3–38.7)
Census Region	
West	20.1 (15.9–25.2)
Midwest	26.0 (21.3–31.2)
South	38.2 (32.9–43.7)
Northeast	15.8 (12.4–19.9)

**Notes:** Data are school-level and take account of the survey design. *n* = 1108 schools from the 2014–2015 School Nutrition and Meal Cost Study (SNMCS). CI: confidence interval.

**Table 2:** Nutritional characteristics of potential competitive entrées regulated by Smart Snacks.

Variable	Mean (95% CI)	IQR
All potential competitive entrées		
Calories (kcal)	307.7 (301.1–314.3)	265.1–348.5
Sodium (mg)	700.5 (686.1–714.9)	604.6–791.3
% of calories from total fat	37.4 (36.9–38.0)	34.4–40.1
% of calories from saturated fat	13.0 (12.6–13.3)	11.1–14.3
% of weight from sugar	4.4 (4.2–4.6)	3.1–5.5

**Notes:** Data are school-level and take account of the survey design. School-level mean nutritional characteristics were computed across all potential competitive entrée items prepared in the given target week. Means and IQRs were computed across schools. *n* = 1108 schools from the 2014–2015 School Nutrition and Meal Cost Study (SNMCS). The nutrient standards for Smart Snacks include: ≤350 calories, ≤480 mg of sodium, ≤35% of calories from total fat, <10% of calories from saturated fat, and ≤35% sugar by weight. CI: confidence interval; IQR: inter-quartile range.

**Table 3:** School-level characteristics associated with the difference in Healthy Eating Index (HEI) score between potential competitive entrées alone and the full reimbursable school lunch.

Variable	Difference in HEI Score
	Coeff. (95% CI)
State Law Requirements	
State law requires schools to follow federal school meal standards	
No ^a^	Referent
Yes	−0.22 (−1.64, 1.19)
State law requires schools to meet or exceed federal Smart Snacks standards for a la carte items	
No ^a^	Referent
Yes	1.20 (−0.47, 2.88)
District Wellness Policy Requirements	
District policy requires schools to follow federal school meal standards	
No ^a^	Referent
Yes	1.64 (−0.10, 3.38)
District policy requires schools to meet or exceed federal Smart Snacks standards for a la carte items	
No ^a^	Referent
Yes	−0.25 (−1.53, 1.02)
School Level	
Elementary school	Referent
Middle school	0.87 (−0.19, 1.93)
High school	0.48 (−0.72, 1.69)
School Offers Universal Free Lunch	
Yes	0.08 (−1.65, 1.82)
No	Referent
Race/Ethnicity of Students in School	
≥50% Non-Hispanic White	Referent
≥50% Non-Hispanic Black	−1.28 (−3.21, 0.65)
≥50% Hispanic	−0.30 (−2.41, 1.80)
Mixed	−0.63 (−2.13, 0.88)
School-level Free/Reduced-Price Lunch Eligibility Rate Tertiles	
Low (0.00–37.42)	Referent
Medium (>37.42–63.37)	−1.57 * (−3.08, −0.06)
High (>63.37–100.00)	−0.88 (−2.72, 0.97)
School Size	
Small (fewer than 500 students)	−1.40 * (−2.79, −0.01)
Medium (500 to 999 students)	0.23 (−0.96, 1.43)
Large (1000 or more students)	Referent
School Urbanicity	
Urban	Referent
Suburban	−0.53 (−1.99, 0.92)
Rural	−1.74 * (−3.41, −0.08)
Census Region	
West	Referent
Midwest	0.47 (−1.56, 2.51)
South	0.30 (−1.50, 2.10)
Northeast	3.14 ** (1.08, 5.20)
Constant	−28.89 *** (−31.52, −26.27)
Adjusted mean difference in HEI score	−28.87

**Notes:** Data are school-level and take account of the survey design. *n* = 1108 schools from the 2014–2015 School Nutrition and Meal Cost Study (SNMCS). HEI-2010 scores for the school lunch were computed for each school based on the weekly average menu prepared. HEI-2010 scores for potential competitive entrée items were computed for each item and then averaged across all items prepared by the school in each school’s given target week. The outcome variable of the linear regression model corresponds to the difference between the HEI-2010 scores for potential competitive entrée items and the school lunch. The adjusted mean difference is adjusted for the variables shown. ^a^ No state law or district policy includes schools that are in states (or districts) with no law on the topic or a weak law that only encourages/suggest meeting the standards. CI: confidence interval. * *p* < 0.05, ** *p* < 0.01 ***, *p* < 0.001.

**Table 4:** School-level characteristics associated with the difference in selected HEI scores between potential competitive entrées alone and the full reimbursable school lunch.

Variable	Total Vegetables	Whole Grains	Protein Foods	Sodium
	Coeff. (95% CI)	Coeff. (95% CI)	Coeff. (95% CI)	Coeff. (95% CI)
State Law Requirements				
State law requires schools to follow federal school meal standards				
No ^a^	Referent	Referent	Referent	Referent
Yes	−0.06 (−0.32, 0.21)	−0.43 (−1.01, 0.15)	0.15 (−0.04, 0.34)	−0.11 (−0.57, 0.36)
State law requires schools to meet or exceed federal Smart Snacks standards for a la carte items				
No ^a^	Referent	Referent	Referent	Referent
Yes	0.18 (−0.19, 0.56)	0.71 * (0.05, 1.37)	−0.11 (−0.32, 0.11)	−0.25 (−0.82, 0.32)
District Wellness Policy Requirements				
District policy requires schools to follow federal school meal standards				
No ^a^	Referent	Referent	Referent	Referent
Yes	0.11 (−0.21, 0.43)	−0.26 (−0.91, 0.39)	−0.08 (−0.30, 0.13)	0.24 (−0.45, 0.93)
District policy requires schools to meet or exceed federal Smart Snacks standards for a la carte items				
No ^a^	Referent	Referent	Referent	Referent
Yes	−0.16 (−0.40, 0.08)	−0.13 (−0.56, 0.31)	0.01 (−0.16, 0.17)	0.41 * (0.01, 0.81)
School Level				
Elementary school	Referent	Referent	Referent	Referent
Middle school	0.27 ** (0.08, 0.47)	−0.08 (−0.39, 0.24)	0.08 (−0.06, 0.22)	−0.08 (−0.41, 0.24)
High school	0.23 * (0.03, 0.43)	−0.03 (−0.41, 0.35)	0.12 (−0.02, 0.26)	0.10 (−0.27, 0.48)
School Offers Universal Free Lunch				
Yes	0.04 (−0.30, 0.39)	0.02 (−0.48, 0.52)	−0.08 (−0.34, 0.18)	0.23 (−0.34, 0.80)
No	Referent	Referent	Referent	Referent
Race/Ethnicity of Students in School				
≥50% Non-Hispanic White	Referent	Referent	Referent	Referent
≥50% Non-Hispanic Black	0.46 (−0.00, 0.92)	−0.29 (−1.05, 0.46)	0.09 (−0.23, 0.42)	−0.94 ** (−1.55, −0.33)
≥50% Hispanic	0.01 (−0.42, 0.45)	0.05 (−0.52, 0.62)	−0.13 (−0.41, 0.14)	−0.63 (−1.35, 0.09)
Mixed	0.13 (−0.21, 0.48)	0.39 (−0.11, 0.90)	−0.07 (−0.33, 0.18)	−0.57 * (−1.07, −0.07)
School-level Free/Reduced-Price Lunch Eligibility Rate Tertiles				
Low (0.00–37.42)	Referent	Referent	Referent	Referent
Medium (>37.42–63.37)	−0.30 * (−0.53, −0.07)	0.02 (−0.43, 0.48)	−0.14 (−0.32, 0.04)	0.28 (−0.14, 0.70)
High (>63.37–100.00)	−0.33 (−0.67, 0.02)	−0.17 (−0.75, 0.40)	−0.20 (−0.43, 0.03)	0.49 (−0.05, 1.03)
School Size				
Small (fewer than 500 students)	−0.42 ** (−0.72, −0.13)	−0.35 (−0.88, 0.18)	−0.03 (−0.21, 0.14)	0.08 (−0.37, 0.52)
Medium (500 to 999 students)	−0.06 (−0.32, 0.20)	0.18 (−0.21, 0.57)	−0.03 (−0.19, 0.14)	−0.02 (−0.43, 0.39)
Large (1000 or more students)	Referent	Referent	Referent	Referent
School Urbanicity				
Urban	Referent	Referent	Referent	Referent
Suburban	0.06 (−0.26, 0.39)	−0.34 (−0.79, 0.12)	−0.11 (−0.36, 0.13)	0.08 (−0.49, 0.64)
Rural	−0.46 ** (−0.80, −0.12)	−0.78 ** (−1.33, −0.23)	−0.05 (−0.30, 0.21)	0.63 * (0.04, 1.23)
Census Region				
West	Referent	Referent	Referent	Referent
Midwest	−0.26 (−0.61, 0.09)	0.06 (−0.65, 0.77)	0.02 (−0.22, 0.26)	0.33 (−0.24, 0.89)
South	−0.06 (−0.43, 0.31)	−0.58 (−1.27, 0.11)	0.03 (−0.22, 0.27)	0.26 (−0.28, 0.80)
Northeast	0.02 (−0.40, 0.44)	0.15 (−0.58, 0.88)	−0.01 (−0.28, 0.25)	0.57 (−0.13, 1.27)
Constant	−2.21 *** (−2.75, −1.67)	−0.51 (−1.42, 0.41)	−0.20 (−0.54, 0.14)	−1.96 *** (−2.85, −1.08)
Adjusted mean difference in HEI score	−2.66	−1.58	−0.42	−1.04

**Notes:** Data are school-level and take account of the survey design. *n* = 1108 schools from the 2014–2015 School Nutrition and Meal Cost Study (SNMCS). HEI-2010 scores for the school lunch were computed for each school based on the weekly average menu prepared. HEI-2010 scores for potential competitive entrée items were computed for each item and then averaged across all items prepared by the school in each school’s given target week. The outcome variable of the linear regression models corresponds to the difference between the HEI-2010 scores for potential competitive entrée items and the school lunch. The adjusted mean differences are adjusted for the variables shown. ^a^ No state law or district policy includes schools that are in states (or districts) with no law on the topic or a weak law that only encourages/suggest meeting the standards. CI: confidence interval. * *p* < 0.05, ** *p* < 0.01, *** *p* < 0.001

## References

[1] United States Department of Agriculture E.R.S. National School Lunch Program. https://www.ers.usda.gov/topics/food-nutrition-assistance/child-nutrition-programs/national-school-lunch-program.aspx.

[2] Food Research and Action Center (FRAC) Facts: National School Lunch Program. https://frac.org/wp-content/uploads/cnnslp.pdf.

[3] U.S. Department of Agriculture, Food and Nutrition Service, Office of Policy Support (2019). School Nutrition and Meal Cost Study, Final Report Volume 1: School Meal Program Operations and School Nutrition Environments.

[4] Hoffman J.A., Rosenfeld L., Schmidt N., Cohen J.F., Gorski M., Chaffee R., Smith L., Rimm E.B. (2015). Implementation of competitive food and beverage standards in a sample of Massachusetts schools: The NOURISH study (Nutrition Opportunities to Understand Reforms Involving Student Health). J. Acad. Nutr. Diet..

[5] Gorski M.T., Cohen J.F., Hoffman J.A., Rosenfeld L., Chaffee R., Smith L., Rimm E.B. (2016). Impact of nutrition standards on competitive food quality in Massachusetts middle and high schools. Am. J. Public Health.

[6] Larson N., Story M. (2010). Are ‘competitive foods’ sold at school making our children fat?. Health Aff..

[7] Cohen J.F., Findling M.T.G., Rosenfeld L., Smith L., Rimm E.B., Hoffman J.A. (2018). The impact of 1 year of healthier school food policies on students’ diets during and outside of the school day. J. Acad. Nutr. Diet..

[8] Healthy, Hunger-Free Kids Act of 2010. Public Law 111–296, 124 Stat, 3183.

[9] United States Department of Agriculture (2016). Local school wellness policy implementation under the Healthy, Hunger-Free Kids Act of 2010: Final rule. Fed. Regist..

[10] United States Department of Agriculture (2012). Nutrition standards in the National School Lunch and School Breakfast Programs; Final rule. Fed. Regist..

[11] United States Department of Agriculture (2016). National School Lunch Program and School Breakfast Program: Nutrition standards for all foods sold in school as required by the Healthy, Hunger-Free Kids Act of 2010. Fed. Regist..

[12] U.S. Department of Health and Human Services, US Department of Agriculture (2010). Dietary Guidelines for Americans.

[13] United States Department of Agriculture A Guide to Smart Snacks in School: For School Year 2019–2020. https://fns-prod.azureedge.net/sites/default/files/resource-files/USDASmartSnacks_508_62019.pdf.

[14] U.S. Department of Agriculture, Food and Nutrition Service, Office of Policy Support (2019). School Nutrition and Meal Cost Study, Final Report Volume 2: Nutritional Characteristics of School Meals.

[15] Cohen J.F., Gorski M.T., Hoffman J.A., Rosenfeld L., Chaffee R., Smith L., Catalano P.J., Rimm E.B. (2016). Healthier standards for school meals and snacks: Impact on school food revenues and lunch participation rates. Am. J. Prev. Med..

[16] U.S. Department of Agriculture, Food and Nutrition Service, Office of Policy Support (2019). School Nutrition and Meal Cost Study, Final Report Volume 4: Student Participation, Satisfaction, Plate Waste, and Dietary Intakes.

[17] Cohen J.F., Richardson S., Parker E., Catalano P.J., Rimm E.B. (2014). Impact of the new US Department of Agriculture school meal standards on food selection, consumption, and waste. Am. J. Prev. Med..

[18] Schwartz M.B., Henderson K.E., Read M., Danna N., Ickovics J.R. (2015). New school meal regulations increase fruit consumption and do not increase total plate waste. Child. Obes..

[19] Cullen K.W., Chen T.-A., Dave J.M. (2015). Changes in foods selected and consumed after implementation of the new National School Lunch Program meal patterns in southeast Texas. Prev. Med. Rep..

[20] Turner L., Leider J., Piekarz-Porter E., Chriqui J.F. (2020). Association of state laws regarding snacks in US schools with students’ consumption of solid fats and added sugars. JAMA Netw. Open.

[21] Chriqui J.F., Lin W., Leider J., Shang C., Perna F.M. (2020). The harmonizing effect of Smart Snacks on the association between state snack laws and high school students’ fruit and vegetable consumption, United States—2005–2017. Prev. Med..

[22] Taber D.R., Chriqui J.F., Powell L.M., Perna F.M., Robinson W.R., Chaloupka F.J. (2015). Socioeconomic differences in the association between competitive food laws and the school food environment. J. Sch. Health.

[23] United States Department of Agriculture (2018). Child nutrition programs: Flexibilities for milk, whole grains, and sodium requirements; Final rule. Fed. Regist..

[24] United States Department of Agriculture (2020). Simplifying meal service and monitoring requirements in the National School Lunch and School Breakfast Programs. Fed. Regist..

[25] United States Department of Agriculture (2020). USDA Announces School and Summer Meals Reforms.

[26] Lott M., Miller L., Arm K., Story M. Rapid Health Impact Assessment on USDA Proposed Changes to School Nutrition Standards. https://healthyeatingresearch.org/.

[27] Gearan E.C., Fox M.K. (2020). Updated nutrition standards have significantly improved the nutritional quality of school lunches and breakfasts. J. Acad. Nutr. Diet..

[28] U.S. Department of Agriculture, Food and Nutrition Service, Office of Policy Support (2019). School Nutrition and Meal Cost Study: Study Design, Sampling, and Data Collection.

[29] Piekarz-Porter E., Chriqui J., Schermbeck R., Leider J., Lin W. (2017). The Active Role States Have Played in Helping to Transform the School Wellness Environment Through Policy, School Years 2006–07 Through 2014–15.

[30] Piekarz-Porter E., Schermbeck R., Leider J., Young S.K., Chriqui J.F. (2017). Working on Wellness: How Aligned Are District Wellness Policies with the Soon-To-Be Implemented Federal Wellness Policy Requirements?.

[31] Guenther P.M., Casavale K.O., Reedy J., Kirkpatrick S.I., Hiza H.A., Kuczynski K.J., Kahle L.L., Krebs-Smith S.M. (2013). Update of the healthy eating index: HEI-2010. J. Acad. Nutr. Diet..

[32] U.S. Department of Education Public Elementary/Secondary School Universe Survey, 2011–2012. https://nces.ed.gov/ccd/pubschuniv.asp.

[33] U.S. Census Bureau Census Regions and Divisions of the United States. http://www2.census.gov/geo/pdfs/maps-data/maps/reference/us_regdiv.pdf.

[34] Turner L., Leider J., Piekarz-Porter E., Schwartz M.B., Merlo C., Brener N., Chriqui J.F. (2018). State laws are associated with school lunch duration and promotion practices. J. Acad. Nutr. Diet..

[35] Chriqui J.F., Turner L., Taber D.R., Chaloupka F.J. (2013). Association between district and state policies and US public elementary school competitive food and beverage environments. JAMA Pediatrics.

[36] United States Department of Agriculture Community Eligibility Provision. https://www.fns.usda.gov/school-meals/community-eligibility-provision.

[37] Hecht A.A., Pollack Porter K.M., Turner L. (2020). Impact of the Community Eligibility Provision of the Healthy, Hunger-Free Kids Act on student nutrition, behavior, and academic outcomes: 2011–2019. Am. J. Public Health.

[38] Briefel R.R., Crepinsek M.K., Cabili C., Wilson A., Gleason P.M. (2009). School food environments and practices affect dietary behaviors of US public school children. J. Am. Diet. Assoc..

[39] Cohen J.F., Lehnerd M.E., Houser R.F., Rimm E.B. (2017). Dietary Approaches to Stop Hypertension diet, weight status, and blood pressure among children and adolescents: National Health and Nutrition Examination Surveys 2003–2012. J. Acad. Nutr. Diet..

[40] Berz J.P., Singer M.R., Guo X., Daniels S.R., Moore L.L. (2011). Use of a DASH food group score to predict excess weight gain in adolescent girls in the National Growth and Health Study. Arch. Pediatrics Adolesc. Med..

[41] Liese A.D., Bortsov A., Günther A.L., Dabelea D., Reynolds K., Standiford D.A., Liu L., Williams D.E., Mayer-Davis E.J., D’Agostino Jr R.B. (2011). Association of DASH diet with cardiovascular risk factors in youth with diabetes mellitus: The SEARCH for Diabetes in Youth study. Circulation.

[42] Barnes T.L., Crandell J., Bell R., Mayer-Davis E., Dabelea D., Liese A. (2013). Change in DASH diet score and cardiovascular risk factors in youth with type 1 and type 2 diabetes mellitus: The SEARCH for Diabetes in Youth Study. Nutr. Diabetes.

[43] Günther A.L., Liese A.D., Bell R.A., Dabelea D., Lawrence J.M., Rodriguez B.L., Standiford D.A., Mayer-Davis E.J. (2009). Association between the dietary approaches to hypertension diet and hypertension in youth with diabetes mellitus. Hypertension.

[44] Saneei P., Hashemipour M., Kelishadi R., Esmaillzadeh A. (2014). The Dietary Approaches to Stop Hypertension (DASH) diet affects inflammation in childhood metabolic syndrome: A randomized cross-over clinical trial. Ann. Nutr. Metab..

[45] Kenney E.L., Barrett J.L., Bleich S.N., Ward Z.J., Cradock A.L., Gortmaker S.L. (2020). Impact of the Healthy, Hunger-Free Kids Act on obesity trends: Study examines impact of the Healthy, Hunger-Free Kids Act of 2010 on childhood obesity trends. Health Aff..

[46] Jia J., Moore L.L., Cabral H., Hanchate A., LaRochelle M.R. (2020). Changes to dietary and health outcomes following implementation of the 2012 updated US Department of Agriculture school nutrition standards: Analysis using National Health and Nutrition Examination Survey, 2005–2016. Public Health Nutr..

[47] Asada Y., Mitric S., Chriqui J.F. (2020). Addressing equity in rural schools: Opportunities and challenges for school meal standards implementation. J. Sch. Health.

[48] Shanafelt A., Hearst M.O., Wang Q., Nanney M.S. (2016). Food insecurity and rural adolescent personal health, home, and academic environments. J. Sch. Health.

